# What to scale first? A cross-sectional analysis of factors affecting cesarean delivery rates at first referral units in Bihar, India

**DOI:** 10.1080/16549716.2023.2202465

**Published:** 2023-05-03

**Authors:** Anna Alaska Pendleton, Rohini Dutta, Minal Shukla, Anusha Jayaram, Anita Gadgil, Sasmita Hembram, Nobhojit Roy, Nakul P. Raykar

**Affiliations:** a Program in Global Surgery and Social Change, Department of Global Health and Social Medicine, Harvard Medical School, Boston, MA, USA; b Department of Vascular and Endovascular Surgery, Massachusetts General Hospital Boston, Boston, MA, USA; c World Health Organization Collaborating Center for Research in Surgical Care Delivery in Low-and-Middle Income Countries, Mumbai, India; d Care India, Patna, India; e Tufts University School of Medicine, Boston, MA, USA; f Department of Public Health Systems, Karolinska Institute, Stockholm, Sweden; g Division of Trauma, Emergency Surgery, Surgical Critical Care, Department of Surgery, Brigham and Women’s Hospital, Boston, MA, USA

**Keywords:** Cesarean delivery, task-sharing, India, obstetric capacity, EmOC

## Abstract

**Background:**

Low rates of caesarean delivery (CD) (<10%) hinder access to a lifesaving procedure for the most vulnerable populations in low-resource settings, but there is a paucity of data regarding which factors contribute most to CD rates.

**Objectives:**

We aimed to determine caesarean delivery rates at Bihar’s first referral units (FRUs) stratified by facility level (regional, sub-district, district). The secondary aim was to identify facility-level factors associated with caesarean delivery rates.

**Methods:**

This cross-sectional study used open-source national datasets from government FRUs in Bihar, India, from April 2018–March 2019. Multivariate Poisson regression analysed association of infrastructure and workforce factors with CD rates.

**Results:**

Of 546,444 deliveries conducted at 149 FRUs, 16961 were CDs, yielding a state-wide FRU CD of 3.1%. There were 67 (45%) regional hospitals, 45 (30%) sub-district hospitals, and 37 (25%) district hospitals. Sixty-one percent of FRUs qualified as having intact infrastructure, 84% had a functioning operating room, but only 7% were LaQshya (Labour Room Quality Improvement Initiative) certified. Considering workforce, 58% had an obstetrician-gynaecologist (range 0–10), 39% had an anaesthetist (range 0–5), and 35% had a provider trained in Emergency Obstetric Care (EmOC) (range 0–4) through a task-sharing initiative. The majority of regional hospitals lack the essential workforce and infrastructure to perform CDs. Multivariate regression including all FRUs performing deliveries demonstrated that presence of a functioning operating room (IRR = 21.0, 95%CI 7.9–55.8, p < 0.001) and the number of obstetrician-gynaecologists (IRR = 1.3, 95%CI 1.1–1.4, p = 0.001) and EmOCs (IRR = 1.6, 95%CI 1.3–1.9, p < 0.001) were associated with facility-level CD rates.

**Conclusion:**

Only 3.1% of the institutional childbirths in Bihar’s FRUs were by CD. The presence of a functional operating room, obstetrician, and task-sharing provider (EmOC) was strongly associated with CD. These factors may represent initial investment priorities for scaling up CD rates in Bihar.

## Introduction

The World Health Organization (WHO) estimates that 300,000 women die during childbirth each year, with 99% of those women living within low- and middle-income countries (LMICs) [1]. Caesarean delivery represents an essential health system component for the prevention of maternal and foetal morbidity and mortality [2]. However, many rural areas experience caesarean delivery rates far below the recommendations secondary to insufficient workforce, infrastructure, and financing [3]. Further investment in obstetric services is a priority for mothers delivering in rural areas to allow access to safe and affordable emergency caesarean delivery by trained healthcare professionals [4].

Bihar, India’s third most populous state with a population of over 100 million, has a maternal mortality rate (MMR) of 149 maternal deaths per 100,000 live births in 2016 [5]. This rate also significantly exceeds the national MMR for India of 113 maternal deaths per 100,000 live births [5]. The state-wide caesarean section rate in Bihar approaches the WHO recommendations at 9.7% [6,7]. However, three-quarters of institutional deliveries occur at government facilities in Bihar, of which caesarean delivery rates approach only 3.6% in these free-for-service government facilities per the National Family Health Survey 5 (NFHS-5) [7]. Prior research has shown that caesarean delivery rates in India are approximately ten times higher in private healthcare facilities as compared to government-owned facilities, yet women from low socio-economic backgrounds often do not have access to caesarean delivery in private facilities due to prohibitively high out-of-pocket pregnancy-related expenses [8–10]. With 88.7% of Bihar’s population living in rural areas and lacking monetary support to access private healthcare facilities, investment in public government healthcare institutions is essential [5].

**Figure 1.**
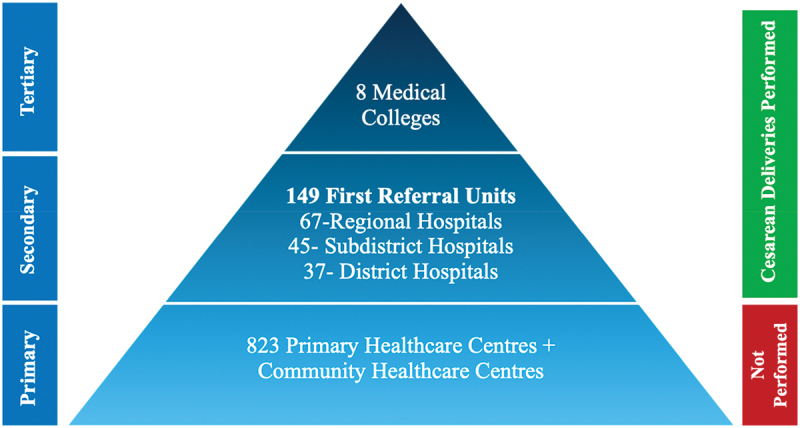
Bihar government-owned health facility structure.

Government-owned facilities in Bihar function at three levels (Figure 1). At the primary level, primary and community health centres function as the first point of patient contact. These facilities lack surgical capacity and are operated by general physicians. At the secondary level, First Referral Units (FRUs) are comprehensive emergency obstetric and newborn care units with the capacity to perform caesarean deliveries. These are further stratified into regional, sub-district and district hospitals. Regional hospitals are the smallest facilities with approximately 30 beds providing healthcare to a population of 80,000 to 120,000. Sub-district hospitals serve a population of 100,000 to 500,000 and have 31–100 beds, and district hospitals provide healthcare services to a population of up to 3,000,000 and have 100–500 beds available. Finally, at the tertiary level, Bihar has eight medical colleges with full surgical and obstetrical capacity.

Factors such as the availability of a skilled obstetrician and anaesthetist and the distance of patient residence from the healthcare facility are known to broadly influence caesarean delivery rates within India [11,12]. Additionally, two task-sharing curricula implemented in Bihar over the past two decades – Emergency Obstetric Care (EmOC) and the Life Saving Anesthesia Skills program (LSAS) – have been shown to impact emergency obstetric procedure rates [13,14]. With primary level facilities not performing caesarean deliveries, FRUs represent the gateway to obstetrical care for Bihar’s predominantly rural population. Although studies have analysed caesarean rates at regional and state levels, there is a paucity of granular data on caesarean operations being performed specifically at FRUs [15,16]. The primary aim of this study was to identify caesarean delivery rates at Bihar’s FRUs stratified by hospital level (regional, sub-district, district). Additionally, there is a lack of data regarding which factors contribute most to caesarean delivery rates at government facilities in Bihar. The secondary aim of this study was to identify facility-level factors associated with caesarean delivery rates at Bihar’s FRUs in order to identify initial maternal healthcare system investment priorities.

## Methods

### Study design

Multiple publicly available datasets were combined to examine facility caesarean rates, infrastructure, and workforce at Bihar’s FRUs stratified by regional, sub-district and district hospitals as defined by the Indian Public Health Standards [17–19]. Facility-level rates of caesarean delivery were obtained from the India Health Management Information Systems (HMIS), an online portal reporting health indicators from state and district-level health authorities, the National Family Health Survey (NFHS), and the District Level Household Survey [7,20]. We collected facility-level data on the number of functioning labour rooms and operating rooms in accordance with guidelines established by the government of India and assessed the presence of blood banks and blood storage units, the number of on-staff obstetrician-gynaecologists, general surgeons, paediatricians, and anaesthetists from data collected from the Maternal Programme Cell, State Health Society of Bihar’s district survey data [21,22]. Data regarding facility infrastructure was collected from the Bihar Medical Services and Infrastructure Corporation (BMSCL) under the aegis of the State Health Society of Bihar [22,23]. The number of emergency obstetric care providers (EmOC) and life-saving anaesthesia skills providers (LSAS) on-staff at each facility was obtained through a dataset from the Training Cell of the National Health Mission of Bihar, a government department responsible for training curricula and record-keeping related to maternal health, family planning, and other programmes [24]. We obtained data regarding the presence of blood banks from the Blood Cell of the State Health Society of Bihar [22]. Any missing data were updated and ratified by the Blood Bank officer-in-charge at the State Resource Unit of Bihar. The facility infrastructure and blood bank status were benchmarked by the Indian Public Health Standards of the National Health Mission [25]. Data were obtained both for fully functional blood banks and for blood storage units (BSUs). Finally, the presence of LaQshya (Labour Room Quality Improvement Initiative) certification, an Indian Ministry of Health and Family Welfare initiative to improve labour room and maternal operating room quality was ascertained for all facilities from records kept by the Quality Cell of the National Health Mission of Bihar [26].

### Data analysis

We calculated descriptive statistics including the caesarean delivery rates at all FRUs, the mean number of facility personnel and proportion of facilities with functioning labour rooms, operating rooms, blood banks or BSUs, and LaQshya certification for all FRUs operating in Bihar over the study period. Results were further stratified by facility level (regional vs sub-district vs district hospital). Poisson regression was performed to analyse the association of all potential infrastructure and workforce covariates with caesarean delivery rates. Only FRUs performing > 0 deliveries over the study period were included in the regression. Covariates were retained in the model unless demonstrating collinearity indicated by a variance inflation factor >10. All analyses were performed using the Stata Version 16 (StataCorp) with robust standard errors and significance defined at a Bonferroni-adjusted alpha-level of 0.005.

## Results

### Description of facilities

In total, 149 government facilities were in operation between April 2018–March 2019. Of these, 67 (45%) were regional hospitals, 45 (30%) were sub-district hospitals, and 37 (25%) were district hospitals (Table 1).

**Table 1.** FRU characteristics stratified by facility level.

Characteristic	Regional Hospitals n (%) Mean (SD)	Sub-District Hospitals n (%) Mean (SD)	District Hospital n (%) Mean (SD)	All Facilities n (%) Mean (SD)
Overall:	67 (45%)	45 (30%)	37 (25%)	149 (100%)
Infrastructure Covariates:				
Intact Infrastructure	22 (33%)	40 (89%)	29 (78%)	91 (61%)
Functioning Labor Room	61 (91%)	43 (96%)	37 (100%)	141 (95%)
Functioning Operating Room	52 (78%)	36 (80%)	37 (100%)	125 (84%)
LaQshya Certification*	0 (0%)	2 (4%)	9 (24%)	11 (7%)
Blood Availability:				
Functioning Blood Bank	0 (0%)	0 (0%)	28 (76%)	28 (19%)
Functioning Blood Bank or BSU	0 (0%)	3 (7%)	36 (97%)	39 (26%)
Number of Cesarean Deliveries	527	1,550	14,884	16,961
% Deliveries via Cesarean	**0.3%**	**1.2%**	**5.7%**	**3.1%**

*LaQshya (Labour Room Quality Improvement Initiative) certification, an Indian Ministry of Health and Family Welfare initiative to improve labor room and maternal operating room quality, was ascertained for all facilities from records kept by the Quality Cell of the National Health Mission of Bihar [27].

Overall, the majority of facilities (61%) were characterised as having good infrastructure (intact building walls and foundation as determined by a BMSCL official), while 39% were characterised as having poor infrastructure. The vast majority of facilities (95%) had functional labour rooms and most facilities (84%) had functional operating rooms. Only 11 facilities (7%) had received LaQshya certification. There were 28 functional blood banks and 11 functional Blood Storage Units (BSUs) between all the sites, for a total of 26% of the facilities with blood capacity. Additional 24 BSUs were proposed, and 53 BSUs were approved but not yet functional.

Substantial disparities existed between facility levels in terms of infrastructure, as demonstrated in Table 1. Overall, district and sub-district hospitals demonstrated higher levels of intact infrastructure and functioning labour rooms and operating rooms than regional hospitals. LaQshya certification was rare among district (24%) and sub-district hospitals (4%), but non-existent at regional hospitals (0%). In terms of blood availability, the vast majority of blood banks and BSUs (36 of 39) were concentrated in district hospitals.

**Figure 2.**
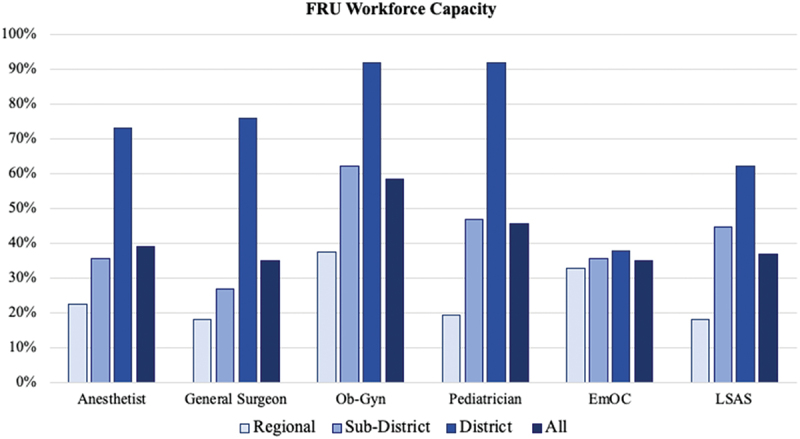
First referral unit (FRU) workforce capacity: percentage of regional, sub-district, and district facilities with at least one on-site care provider in the designated field.

Regarding facility personnel, 58% of the facilities had at least one obstetrician-gynaecologist on staff (range 0–10 obstetrician-gynaecologists), 46% had a paediatrician on staff (range 0–10), 38% had an anaesthetist on staff (range 0–5), and 35% had a general surgeon on staff (range 0–22) (Figure 2). Two task-sharing training programmes were conducted at the facilities prior to the survey. Fifty-two facilities (35%) had a provider trained in EmOC (range 0–4 providers), and 37% of the facilities had providers trained in LSAS (range 0–3). Stratified by facility level, the highest workforce capacity was identified at district hospitals, with these facilities having the highest numbers of on-staff anaesthetists, general surgeons, obstetrician-gynaecologists and paediatricians as well as task-sharing workforces (EmOCs and LSAS).

### Description of deliveries

From April 2018–March 2019, 546,444 deliveries were conducted at FRUs within Bihar. The number of deliveries at each facility ranged from 0 to 17,571 (mean 3,667 ± 3,008 deliveries). In total, 16961 caesarean deliveries were performed, for a state-wide FRU caesarean-delivery rate of 3.1%, although individual facility caesarean delivery rates ranged from 0% to 32.5%. Mean caesarean delivery rates differed between regional (0.3%), sub-district (1.2%), and district facilities (5.7%) (p < 0.005). Seventeen facilities performed no deliveries over the study period, of which 11 were regional hospitals and 6 were sub-district hospitals.

### Poisson regression

Multivariate regression analysis, including all FRUs performing deliveries over the study period (n = 132) demonstrated that the presence of a functioning operating room and the number of obstetrician-gynaecologists and EmOCs were associated with facility-level caesarean delivery rates (p < 0.005) (Table 2).

**Table 2.** Regression analysis of facility factors associated with caesarean delivery rate.

Characteristic	Incidence Rate Ratio (95% CI)^A^	P-Value^B^
Intact Facility Infrastructure	1.25 (0.55, 2.85)	0.589
Facility Level:		
Regional Hospital	-	-
Sub-District Hospital	2.98 (0.67, 13.28)	0.153
District Hospital	4.27 (0.95, 19.19)	0.058
Functioning:		
Operating Room	20.97 (7.88, 55.81)	**< 0.001**
Number of On-Staff:		
Anesthetists	1.15 (0.90, 1.48)	0.267
General Surgeons	0.87 (0.77, 0.98)	**0.020**
Obstetrician-Gynecologists	1.25 (1.09, 1.43)	**0.001**
Pediatricians	1.21 (0.95, 1.56)	0.127
Number of Trained:		
EmOC Providers^C^	1.56 (1.27, 1.92)	**< 0.001**
LSAS Providers^D^	0.83 (0.53, 1.29)	0.410
LaQshya Certification	1.57 (0.76, 3.25)	0.220

^A^Regression analysis performed using robust variances.

^B^Bold values significant at Bonferroni-corrected p-value of 0.005.

^C^Emergency Obstetric Care Providers.

^D^Life Saving Anesthesia Skills Providers.

Facility level (regional vs sub-district vs district), LaQshya certification, number of anaesthetists, general surgeons, paediatricians, and LSASs were not significantly associated with facility delivery rates. Blood bank availability was excluded from the analysis given collinearity with facility level (36/38 facilities with a blood bank or BSU were district hospitals).

## Discussion

This study describes facility-level disparities between the regional, district, and sub-district facilities and identifies factors influencing caesarean delivery rates at FRUs and provides insight into possible areas of focus to improve Bihar’s maternal healthcare system.

### Bihar’s FRU cesarean delivery rate is far below WHO recommendations

FRUs are the gateway to obstetric care for millions living in Bihar, yet the overall FRU caesarean rate of 3.1% is dramatically lower than the statewide, national, and internationally accepted recommended caesarean delivery rate [27]. This rate is consistent with the NFHS-5 2019–2020 data, in which caesarean delivery rates were found to be below 10% in government facilities in four states of India: Mizoram, Meghalaya, Nagaland, and Bihar [7]. However, this rate is far below the national caesarean rate of 17.2% for India as per NFHS-4 [8]. The higher national caesarean rate reflects a large number of caesarean deliveries occurring in private healthcare facilities in urban areas [16]. Prior data indicate that high caesarean delivery rates in the private sector may be associated with profit-driven incentives, resulting in unnecessary, elective caesarean deliveries [28]. The very low caesarean delivery rate occurring in Bihar under austere conditions (sometimes in facilities even without access to blood) indicates that these procedures may be predominantly emergent or urgent in nature and further emphasises the critical need for upscaling of the obstetric care capacity in these rural facilities.

While the overall institutional delivery rates in Bihar have increased from 63% to 75% in the past 5 years, the low caesarean delivery rates in Bihar’s FRUs revealed in this study raise concern, as it leads to decreased access to caesarean delivery for the poorest and most rural populations of the state [7,29]. Moreover, data from Molina et al. suggests that perhaps an even higher caesarean delivery rate, up to 19 per 100 live births, should be targeted to reduce maternal and neonatal mortality [29].

### Cesarean delivery rates differed substantially between regional-, sub-district- and district-level facilities

Stratified analysis revealed that Bihar’s overall FRU caesarean delivery rate did not represent homogeneous conditions across the state, but rather that significant disparities exist between regional, sub-district and district hospitals. Though all facility-level caesarean delivery rates were below WHO recommendations, district hospitals most closely approached this target with a caesarean delivery rate of 5.7%, while sub-district (1.2% caesarean delivery rate) and regional hospitals (0.3%) fell substantially short of this aim. FRUs are in part defined by their ability to conduct caesarean deliveries, yet stratification demonstrated stark disparities in obstetric capacity and revealed that regional hospitals lacked resources essential to performing caesarean deliveries [21]. For example, while 100% of district hospitals had a functional labour room and operating room, 22% of the regional hospitals had no functional operating room and 9% had no functioning labour room. By demonstrating significant disparities between FRUs, stratified analysis revealed the error of implementing interventions broadly across all FRU facility levels. Whereas, the largest district hospitals in most cases have adequate resources to perform caesarean delivery in the case of obstetric emergency, smaller sub-district and district hospitals lack obstetric care essentials in terms of workforce and infrastructure. Future initiatives to address low caesarean rates in Bihar must consider the FRU levels at which workforce and infrastructure improvements are needed most to ensure that resources are distributed the most equitably and productively.

Specifically, considering workforce capacity, only 37% of the regional hospitals had at least one obstetrician-gynaecologist, as compared to 62% of the sub-district hospitals, and 92% of district hospitals. It is likely that low caesarean delivery rates at FRUs in Bihar, particularly regional facilities, reflects a lack of adequate resources to handle challenging obstetric emergencies, resulting in the transfer of patients to higher-level facilities. Data from a tertiary care centre in Jodhpur indicate that primary reasons for patient referral were the non-availability of essential workforce (including obstetricians and anaesthetists) trained to manage obstetric emergencies and the lack of facilities capable of performing caesarean deliveries [30]. Unfortunately, referral of obstetric emergencies to higher levels of care is not without adverse patient outcomes. In Madhya Pradesh, a poor referral system for obstetric emergencies has been demonstrated to lead to an increased maternal mortality [31].

Another significant disparity between FRUs was access to blood. Only 26% of all FRUs had access to a blood bank or BSU, of which 92% were district hospitals and 8% were sub-district hospitals. Although the presence of a blood bank or blood storage unit (BSU) was excluded from the multivariate analysis given collinearity with facility level, previous studies demonstrate a strong correlation between blood availability and provision of caesarean delivery [32]. Caesarean delivery rates were 19-times higher for Bihar’s district hospitals (of which 97% had access to a blood bank or BSU) as compared to regional hospitals (of which 0% had access to a blood bank or BSU). Access to blood likely plays a significant role in providers’ decision to proceed with an invasive, surgical intervention. Further research should assess the impact that lack of access to blood has on provider decision algorithms regarding caesarean delivery.

### Importance of obstetrician-gynecologists and task-sharing workforce

Findings from this study highlight the importance of access to trained obstetrical care providers in increasing access to caesarean delivery. Multivariate regression analysis, including all FRUs performing deliveries over the study period, demonstrated that the number of on-staff obstetrician-gynaecologists was independently associated with facility-level caesarean delivery rates (p < 0.005). This finding reflects the fact that a shortage of the obstetric workforce constitutes an important barrier to safe caesarean delivery. Similar observations have been reported by Ntambue et al., from the Democratic Republic of Congo, as well as from Bruckmann et al. in Pakistan [33,34]. These studies reinforce the importance of investment in human capital and a strong obstetrical workforce. Although in Bihar the presence of on-staff obstetrician-gynaecologists was associated with increasing facility caesarean delivery rates, only 58% of the facilities had at least one obstetrician-gynaecologist on staff. In recent years, the private sector share in obstetric services has grown significantly in India despite higher involved expenses [35]. India, like most LMICs, faces health workforce shortages and difficulties in recruiting and retaining trained EmOCs and obstetricians in rural and remote areas [36]. This shortage of trained obstetrical workforce is a critical barrier to providing essential obstetrical services to Bihar’s population.

One possible method of meeting the obstetrical workforce needs in Bihar is through task-sharing programmes. This study demonstrates that the presence of at least one Emergency Obstetric Care (EmOC) provider significantly increased facility caesarean delivery rates (IRR 1.56, p < 0.001). The EmOC curriculum is a 6-month course for MBBS physicians that teaches emergency obstetric care in the absence of a certified obstetrician-gynaecologist [13]. This study suggests that EmOC training is an effective way of increasing the workforce trained to perform caesarean deliveries in Bihar. These findings are consistent with data from Ziraba et al.*’s* study of maternal outcomes in Nairobi, Owens et al.*’s* study of emergency obstetric care in Zambia, and Banstola el al.*’s* findings in Nepal [37–39]. Although the presence of a LSAS-trained doctor was not associated with facility caesarean delivery rates in multivariate regression, these clinicians are taught skills to resuscitate and stabilise mothers in order to transfer them to a higher level facility [40]. Therefore, the positive impact of the LSAS curriculum may not be reflected in increased FRU caesarean delivery rates. Taken together, the findings of the present study contribute to the growing literature suggesting that task-sharing may be leveraged to improve access to essential maternal care services in obstetrical emergencies.

### Need for investment in operation rooms to increase caesarean delivery rates

In multivariate regression analysis, the presence of a functional operating room was significantly associated with caesarean delivery rates. Caesarean delivery requires a sterile environment and the availability of surgical instruments. Given the financial investment, workforce, and infrastructure necessary to maintain a functional operating room, it is likely that this covariate will serve as a proxy measure for a facility with robust operative capacity. The presence of a functioning operating room represents a strong investment not only for upscaling maternal and child healthcare services but also for improving the overall facility surgical capacity [41].

Improving infrastructure in low-resource settings has been shown to play a significant role in caesarean delivery outcomes [42]. Poor-quality care contributes to more deaths than a lack of access: nearly twice as many Indians died in 2016 secondary to poor quality of care as compared to those who died from non-utilisation of health services [43]. LaQshya certification (a marker of obstetrical service quality) was absent in the vast majority of FRUs (only 7% of FRUs in Bihar were LaQshya certified). This facility factor was not associated with caesarean rates in multivariate regression. However, the scarcity of LaQshya certification considered with the association of a functional operating room with caesarean delivery rates underscores that in the effort to increase access to caesarean delivery, investment in high-quality operative infrastructure should not be compromised [44].

Although this analysis was limited to a single Indian state, our results highlight the obstetrical care challenges in the third-most populous state of India [5]. With 88.7% of Bihar’s population living in rural areas, this study specifically identifies barriers to high-quality obstetric care at FRUs for India’s rural population [5,45]. Although this study evaluated all FRUs in Bihar, medical colleges, which represent other government facilities capable of performing caesarean deliveries, were not included in this study. We specifically chose to focus on FRUs given that these institutions represent the primary site of maternal care for Bihar’s predominantly rural population. Another limitation of this study is that given the method of data collection, more granular information regarding site-specific factors influencing caesarean delivery rates was not available. For example, one facility in this study reported a greater than 30% caesarean delivery rate. The indications for caesarean delivery, patient demographic and social factors, availability of non-governmental blood, and maternal mortality data were not available for this study. Finally, specifically considering the impact of blood availability on caesarean delivery rates, the current dataset was limited to public sector blood banks and BSUs in Bihar and does not include demand met by private blood banks and non-governmental organisations.

Future work should aim to assess the correlation of FRU caesarean delivery rates and facility-level workforce and infrastructure factors with maternal and infant mortality rates (data not available in the present study). Further qualitative analyses are needed to assess the nuances behind factors driving caesarean deliveries at these facilities and the decision to proceed with caesarean delivery in austere environments.

## Conclusion

Only 3.1% of the institutional childbirths in government facilities at the first referral units in Bihar were by caesarean delivery. In the presence of a functional operating room, obstetricians and task-sharing providers (EmOCs) were strongly associated with caesarean delivery rates. These findings highlight factors that may represent initial investment priorities for scaling up caesarean delivery rates in Bihar. Ultimately, this work highlights the multifaceted challenges in advancing surgical care in rural and low-resource settings and reinforces a growing consensus around the importance of human resources in provision of high-quality maternal healthcare.

## Data Availability

The datasets used and/or analysed during the current study are available from the corresponding author at reasonable request. http://rchiips.org/nfhs/nfhs4.shtml

## Paper context

Low rates of caesarean delivery hinder access for the most vulnerable populations in low-resource settings, but there is a paucity of data regarding factors contributing most to caesarean delivery rates. Only 3.1% of the institutional childbirths in Bihar’s First Referral Units were by caesarean delivery. The presence of a functional operating room, obstetrician, and task-sharing provider was strongly associated with caesarean delivery, which may represent initial investment priorities for scaling up caesarean delivery in Bihar.
